# Supplementary material for: Graph-based open-ended survey on concerns related to COVID-19
Source: PLoS One. 2021 Aug 13;16(8):e0256212. doi: 10.1371/journal.pone.0256212 (PMC8362959; doi:10.1371/journal.pone.0256212)
Supplement: S1 File — (PDF) [file pone.0256212.s001.pdf]

Supplementary Information:
Graph-based open-ended survey on concerns related to COVID-19

Tatsuro Kawamoto
Artificial Intelligence Research Center,
National Institute of Advanced Industrial Science and Technology, Tokyo, Japan

Takaaki Aoki
Faculty of Education, Kagawa university, Takamatsu, Japan

Michiko Ueda
Faculty of Political Science and Economics, Waseda University, Tokyo, Japan

ON THE DEFINITION OF THE OPINION GRAPH

The data collection procedure in this study is essentially the same as the survey conducted in [S1]. However, we considered bipartite opinion graphs, and treated both opinion and respondent vertices as vertices of the same type (i.e. monopartite graph) in [S1]. Note that we only considered the single-edge type in this study for simplicity, whereas we considered multiple-edge types (positive and negative) in [S1] for graph clustering.

INITIAL OPINIONS

Below is a list of the initial opinions that were randomly presented to respondents. The original texts were written in Japanese.

- Domestic violence
- I had to close my business because of a declining number of customers.
- I cannot concentrate on my work because of school closure.
- I am afraid of the pressure that I might experience from others if I become infected with COVID-19.
- My business is suffering.
- I do not know what is going to happen to me if I become infected.
- I am hesitant to visit my doctor even when I feel sick with the common cold, etc., because I do not want to catch the virus.
- I cannot get a PCR test even when I want to get tested.
- I cannot get a paid leave even if I tested positive with COVID-19, and I would have no choice but to take a leave without pay or quit my job.
- I am not sure if I can afford to pay medical bills if I become infected.
- I am spending too much on childcare costs.
- I do not have much money left and I am not sure if I can survive without public assistance.

These opinions were selected because they could be considered typical opinions. We also included several opinions from each of the potential major categories: infection risk, social pressure/future prospect, and financial issues.

We provided these initial opinions to help respondents understand the scope of the question. As pointed out in [S2], providing the scope of a question is important for an open-ended question, and it is often mentioned in the question. If no information about the scope was provided, then the diversity of opinions could be narrower than what could have been obtained.

A drawback of the initial opinions is that they may influence respondents' opinions. However, the contents of the resulting opinion groups sufficiently deviated from the initial opinions; thus, it is unlikely that these initial opinions influenced the results of the present study.

OPINION GROUPS

Brief descriptions of each opinion group are provided below.

infection risk: Concerns related to the infection risk of COVID-19

social pressure & future prospect: Fears that they may be criticised should they be tested positive for COVID-19; concerns about uncertain future prospects

financial issues: Concerns about income and/or employment status

travel: Dissatisfaction with difficulties in traveling or going out

government policies: Issues related to one-time cash handouts, dissatisfaction with politicians

mask (shortage): Shortage of masks at pharmacies

mask (discomfort): Discomfort associated with wearing a mask

other issues: This group contains various valid opinions that are not classified into other opinion groups identified in each survey. For example, we identified the following contents in this opinion group:

1. Worries about the capacity of hospitals
2. Frustration with the behaviour of others, or school
3. Shortage of items in stores (other than masks); physical and mental health
4. Family-related issues (e.g. parenting)

no concerns: No particular concerns about the present situation.

invalid responses: Responses that are not directly related to the question asked in the present survey

CONSISTENCY AMONG THE ANNOTATORS

The consistency among the three annotators is shown in the tables in Fig. S1.

[S1] T. Kawamoto and T. Aoki, Democratic classification of free-format survey responses with a network-based framework, Nature Machine Intelligence **1**, 322 (2019).

[S2] H. Schuman and J. Scott, Problems in the Use of Survey Questions to Measure Public Opinion, Science **236**, 957 (1987).

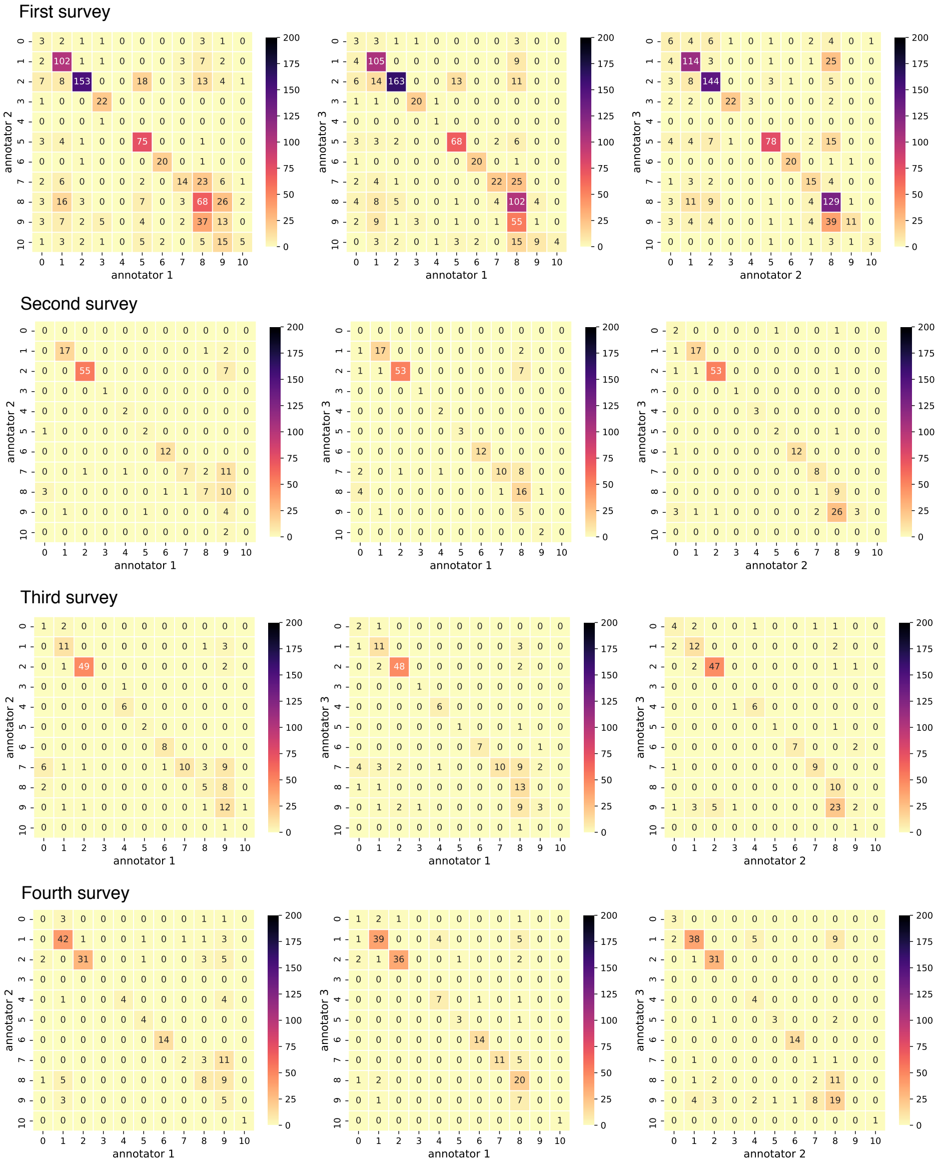

FIG. S1. Agreement between each pair of annotators with respect to the opinion group labels.
