# Supplementary material for: Graph-based open-ended survey on concerns related to COVID-19
Source: PLoS One. 2021 Aug 13;16(8):e0256212. doi: 10.1371/journal.pone.0256212 (PMC8362959; doi:10.1371/journal.pone.0256212)
Supplement: S1 Table — (PDF) [file pone.0256212.s002.pdf]

S1 Table: Demographic Attributes of the Respondents

	All		1st survey		2nd survey		3rd survey		4th survey	
	N	%	N	%	N	%	N	%	N	%
**All**	7007	100.0	2103	100.0	1516	100.0	1729	100.0	1659	100.0
**Sex**										
Male	3928	56.1	1156	55.0	849	56.0	971	56.2	952	57.4
Female	3079	44.0	947	45.0	667	44.0	758	43.8	707	42.6
**Age Groups**										
20-29	1028	14.6	429	20.4	231	15.2	195	11.3	173	10.4
30-39	1460	20.8	498	23.7	301	19.9	349	20.2	312	18.8
40-49	2268	32.4	572	27.2	448	29.6	610	35.3	638	38.5
50-59	2251	32.1	604	28.7	536	35.4	575	33.3	536	32.3
**Occupation/Employment Status**										
Working full-time	3818	54.5	1128	53.6	808	53.3	946	54.7	936	56.4
Housekeeping and working	552	7.9	161	7.7	117	7.7	147	8.5	127	7.7
Student and working	72	1.0	23	1.1	14	0.9	18	1.0	17	1.0
On leave from work	329	4.7	103	4.9	98	6.5	72	4.2	56	3.4
Seeking job	395	5.6	119	5.7	93	6.1	87	5.0	96	5.8
Housekeeping	1235	17.6	362	17.2	263	17.4	323	18.7	287	17.3
Student	141	2.0	47	2.2	35	2.3	36	2.1	23	1.4
Other	465	6.6	160	7.6	88	5.8	100	5.8	117	7.1
